# Non‐Monotonic Ion Conductivity in Lithium‐Aluminum‐Chloride Glass Solid‐State Electrolytes Explained by Cascading Hopping

**DOI:** 10.1002/advs.202509205

**Published:** 2025-09-17

**Authors:** Beomgyu Kang, Jina Yu, Shinji Saito, Jihyun Jang, Bong June Sung

**Affiliations:** ^1^ Department of Chemistry Sogang University Seoul 04107 Republic of Korea; ^2^ Institute for Molecular Science and The Graduate University for Advanced Studies (SOKENDAI) Myodaiji, Aichi Okazaki 444‐8585 Japan; ^3^ Department of Chemistry and Center for Nano Materials Sogang University Seoul 04107 Republic of Korea; ^4^ Department of Chemistry and Institute of Biological Interfaces Sogang University Seoul 04107 Republic of Korea

**Keywords:** cascading hop, dynamic heterogeneity, ion conduction mechanism, solid state electrolyte

## Abstract

Inorganic glass solid‐state electrolytes (IGSSEs) exhibit superionic conductivity at ambient temperature. Understanding their ion conduction mechanism remains challenging but is essential for the development of next‐generation all‐solid‐state batteries. The coupling between lithium ion diffusion and the rotation of neighboring polyanions, known as the paddlewheel effect, has been proposed as a possible mechanism, though its existence remains controversial. Herein, a systematic and extendible approach is proposed to explore the ion conduction mechanism of IGSSEs using large‐scale machine learning molecular dynamics (MLMD) simulations and hop function analysis. A machine learning potential is constructed for model IGSSEs of Li_*x*_AlCl_3 + *x*_ (*x* = 0.25 to 3). MLMD simulation results reproduce the experimentally observed non‐monotonic composition dependence of lithium‐ion conductivity, with a maximum at *x* = 1. Hop function analysis reveals that lithium ion diffusion occurs mainly via cascading hopping events rather than paddlewheel motions. The cascading hops are composition‐dependent and account for the observed non‐monotonic composition dependence. The non‐monotonic composition dependence arises from a delicate balance between the local concentrations of lithium ions and the lithium vacancies.

## Graphical abstract

Cascading hopping events of lithium ions are identified as the main ion conduction mechanism in inorganic glass solid‐state electrolytes. Machine learning molecular dynamics simulations and hop function analysis confirm that pairs of lithium ions carry out cascading hopping motions. This mechanism accounts for the non‐monotonic composition dependence of lithium transport, which cannot be explained by the well‐known paddelwheel mechanism.

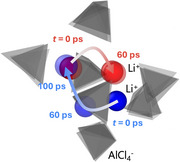

## 1 Introduction

The demand for all‐solid‐state batteries (ASSBs) with improved stability and high energy density grows at an unprecedented rate. Inorganic glass solid‐state electrolytes (IGSSEs) show superionic conductivity at ambient temperature^[^ ^1^, ^2^, ^3^, ^4^ ^]^ and play a pivotal role in dictating the overall electrochemical performances of ASSBs, with ionic conductivity being one of the most critical determinants. Therefore, a comprehensive understanding of lithium ion transport mechanisms within IGSSEs is imperative for further improvement in ionic conductivity^[^ ^5^, ^6^, ^7^, ^8^, ^9^ ^]^ but remains challenging, particularly for dynamically heterogeneous IGSSEs. In this work, we propose a systematic approach for exploring the ion transport mechanism via hop function analysis and large‐scale molecular dynamic (MD) simulations with machine learning potentials (MLPs). This approach allows us to consider the dynamic heterogeneity of lithium ions in IGSSEs at large spatiotemporal scales and evaluate how local environments around lithium ions would correlate with the ion conductivity.

If the Stokes‐Einstein relation (SER), the foundation of equilibrium statistical mechanics, were to hold, the ion diffusion should be inversely proportional to the high viscosity of IGSSEs and is expected to be low.^[^ ^10^, ^11^, ^12^, ^13^ ^]^ The amorphous solids (or glasses), however, often violates the SER such that the diffusion in glasses becomes spatially heterogeneous: some ions may diffuse fast while other ions of even the same kind diffuse slowly.^[^ ^14^, ^15^, ^16^, ^17^, ^18^ ^]^ Such a dynamic heterogeneity can be a key concept to explain the high ion conductivity of IGSSEs. In this study, we illustrate that the diffusion of lithium ions in IGSSEs is significantly heterogeneous, i.e., some lithium ions rattle during nanoseconds within small cages of neighbor polyanions (sustaining the materials in the solid‐state) while other lithium ions perform hopping motions between cages (responsible for high ion conductivity). The hop function analysis suggests that in case of a model IGSSE in this study, a lithium ion hops to a vacancy where its neighbor lithium ions are depleted, thus resulting in cascading hopping motions of neighbor lithium ions.

The effect of a local environment around a lithium ion has been investigated as a transport mechanism. Especially, it has been an issue whether the rotation of polyanions around the lithim ion would enhance its conductivity. The paddlewheel effect (or cog‐wheel effect) of polyanions has been considered an important transport mechanism for crystalline solids such as Li_2_SO_4_.^[^ ^19^, ^20^ ^]^ Fang and Jena suggested that the paddlewheel motion of polyanions would decrease the energy barrier of the lithium diffusion.^[^ ^21^ ^]^ On the other hand, in other crystalline solids of argyrodites with pseudohalogens, Sun et al. reported that the rotational motion of polyanions correlated only weakly with the lithium conductivity.^[^ ^22^ ^]^ Xu et al. carried out MLMD simulations for various solid state electrolytes systematically and showed that not only the paddle‐wheel effect would be weak but also the rotation of some polyanions could impact the lithium ion diffusion even in a negative fashion.^[^ ^23^, ^24^ ^]^ Recent ab initio molecular dynamics (AIMD) studies on crystalline solid electrolytes revealed that Li ions should undergo strongly correlated hopping motions, i.e., concerted migration, which should reduce the energy barriers of Li ion diffusion and facilitate the ion conductivity.^[^ ^25^, ^26^, ^27^, ^28^ ^]^ In case of amorphous sulfide‐based (LPS) systems, whether the polyanions would serve as a paddlewheel remains unlear^[^ ^29^, ^30^, ^31^ ^]^ and there is an ongoing debate over the paddlewheel effect.^[^ ^32^, ^33^ ^]^ In our simulation system, the rotational hopping motions of polyanions occur at the same timescales of the lithium ion hopping motions. However, the hop function analysis suggests that the polyanion rotation would not correlate with lithium ion hopping motions. We find that it is not the polyanion rotation but the dynamic heterogeneity of lithium ions that can explain the complicated composition dependence of the conductivity of lithium ions.

One of the predominant ASSB types employs sulfide‐based solid‐state electrolytes (SSEs) such as Li_2_S·P_2_S_5_ and Li_6_PS_5_Cl, which offer relatively high ionic conductivity, facile manufacturability, and good processability.^[^ ^34^, ^35^ ^]^ However, sulfide SSEs suffer from a limited electrochemical stability window, particularly poor oxidative stability, which presents a significant challenge in integrating high‐voltage cathodes for achieving higher energy densities.^[^ ^36^, ^37^ ^]^ To address this limitation, halide‐based SSEs have been developed as an alternative, leveraging their higher oxidation stability window above 4V (*v*.*s*. Li/Li^+^) and chemical compatibility with cathodes by substituting sulfur anions in sulfide SSEs with halide anions.^[^ ^38^ ^]^ Despite this advantage, halide‐based SSEs generally exhibit lower ionic conductivities, typically below 1 mS cm^−1^, necessitating innovative material design strategies to enhance their ion transport properties. Recent studies have demonstrated that the introduction of structural amorphization or lithium‐ion vacancy engineering in halide SSEs can improve ionic conductivity by more than an order of magnitude.^[^ ^3^, ^39^, ^40^, ^41^ ^]^ However, the extent of these enhancements remains constrained due to an imcomplete understanding of the underlying mechanisms.

There have been efforts to optimize the halide composition of halide‐based SSEs and improve the ion conductivity.^[^ ^42^, ^43^, ^44^ ^]^ A recent experiment for Na_2.25 − *x*_Y_0.25_Zr_0.75_Cl_6 − *x*_ (*x* = 1.375 to 2) showed that the ion conductivity of sodium ions showed a complex non‐monotonic trend with *x*.^[^ ^45^ ^]^ The sodium ion conductivity was maximum at *x* = 1.625 while the activation energy for the conductivity increased monotonically with *x*. If one were to elucidate the ion conduction mechanism that may explain the complex composition dependence of ion conductivity, it would promote the design of IGSSEs with an optimal conductivity. In this study, we focus on a model lithium halide SSE of Li_*x*_AlCl_3 + *x*_ with *x* from 0.25 to 3 and find that the lithium conductivity also shows a non‐monotonic behavior with a maximum at *x* = 1. LiAlCl_4_ has been reported to exhibit an ionic conductivity on the order of 10^−6^ to 10^−5^ Scm^−1^ at room temperature.^[^ ^46^, ^47^, ^48^ ^]^ While this absolute value is relatively low, the ionic conductivity could be enhanced to the 10^−3^ Scm^−1^ range through the optimization of synthesis conditions and doping with other elements such as oxygen.^[^ ^49^ ^]^ By analyzing how the composition and structural components of Li_*x*_AlCl_3 + *x*_ influence lithium‐ion transport mechanisms and conductivity, this study provides a valuable model for identifying and designing next‐generation solid‐state electrolytes with enhanced performance suitable for all‐solid‐state batteries. We aim to identify key compositional and structural factors that influence ion mobility in this class of materials.

The investigation of dynamic heterogeneity in amorphous solids requires computational approaches of large spatiotemporal scales. AIMD should be an excellent approach to investigate the local environment and paddlewheel effect of polyanions around lithium ions since the polyanion rotation occurs within tens of picoseconds.^[^ ^50^, ^51^ ^]^ However, when the systems are quite heterogeneous like IGSSEs, we need to consider several domains of different ion mobilities at multiple timescales beyond the limitation of AIMDs and quantify the heterogeneity of the lithium ions.^[^ ^52^ ^]^ MD simulation with MLPs can fill the gap between the accuracy of density functional theories (DFTs) and the efficiency of classical molecular dynamics simulations.^[^ ^53^, ^54^, ^55^ ^]^ MLP has been successfully introduced to simulating SSEs that require large‐scale simulations.^[^ ^56^, ^57^, ^58^, ^59^ ^]^ In this study, we develop MLP to simulate bulk Li_*x*_AlCl_3 + *x*_ at a length and times scales of around 4 nm and nanoseconds, which allows us to investigate the dynamic heterogeneity of the system.

## 2 Results

### 2.1 The Composition Dependence of Ion Conductivity and Dynamic Heterogeneity

Li_*x*_AlCl_3 + *x*_ systems in our simulations are amorphous solids at room temperature, where lithium ions are well dispersed and ${\mathrm{AlCl}}_{4}^{-}$ ions form stable tetrahedra without long‐range periodicity. This shares similar structural features found in amorphous solid lithium sulfide glass.^[^ ^57^ ^]^ Note that while training the MLP, we do not incorporate any structural constraint for ${\mathrm{AlCl}}_{4}^{-}$. Each aluminum ion is coordinated with four chloride ions with an inter‐ion distance of about 2 Å. Each ${\mathrm{AlCl}}_{4}^{-}$ tetrahedron is very stable throughout our simulations. More details for structural aspects of amorphous Li_*x*_AlCl_3 + *x*_ solids are in the Figure S1 (Supporting Information).

**Figure 1:**
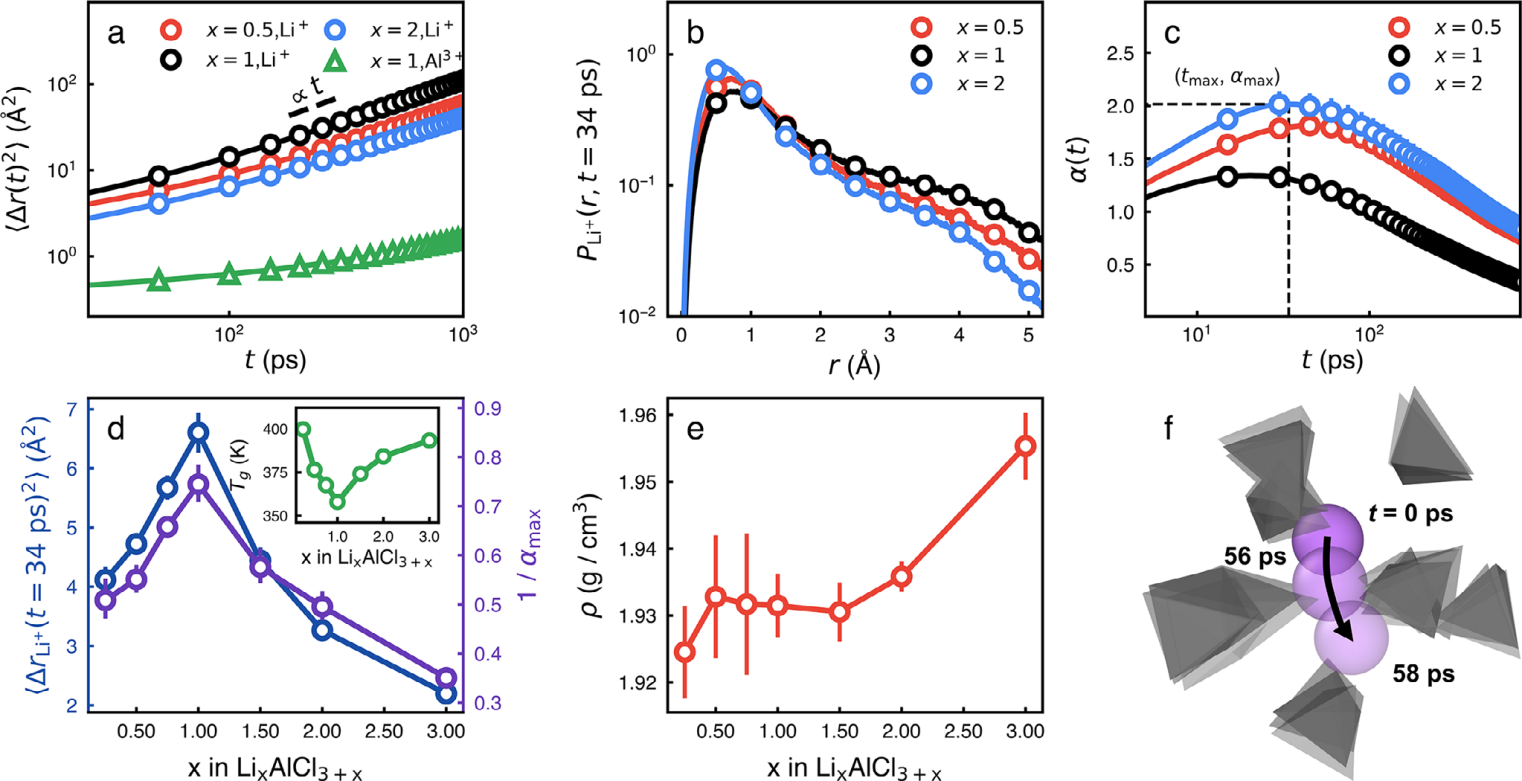
The lithium ion conductivity and its dependence on the composition. a) The mean‐squared displacement (〈Δ*r*(*t*)^2^〉) of lithium and aluminum ions in Li_*x*_AlCl_3 + *x*_ for different values of *x*. Note that in case of aluminum ions, 〈Δ*r*(*t*)^2^〉 is insensitive to *x* and is provided only for *x* = 1. b) The self‐parts of van Hove correlation functions $P_{{\mathrm{Li}}^{+}}\left(r,t=34\;\mathrm{ps}\right)=4\pi r^{2}G_{s}\left(r,t=34\;\mathrm{ps}\right)$ of lithium ions in Li_*x*_AlCl_3 + *x*_ for different values of *x*, illustrating that the lithium ion diffusion is heterogeneous and some lithium ions hop. c) The non‐Gaussian parameters (α(*t*)) of lithium ions in Li_*x*_AlCl_3 + *x*_ for different values of *x*. (*t*_*max*_, α_*max*_) corresponds to the point where α(*t*) is maximum and the dynamic heterogeneity is the most significant.d) The mobility *M* (≡ 〈Δ*r*(*t* = 34 ps)^2^〉 and the inverse of α_*max*_ of lithium ions as a function of *x*. In the inset is the glass transition temperature (*T*_*g*_) of Li_*x*_AlCl_3 + *x*_ as a function of *x*. e) The density (ρ) of Li_*x*_AlCl_3 + *x*_ as a function of *x*. f) The representative trajectory of a lithium ion (purple sphere) that undergoes hopping motions. Grey tetrahedra represent neighbor ${\mathrm{AlCl}}_{4}^{-}$ polyanions.

Lithium ions are diffusive at timescales of 10 ns while tetrahedra of ${\mathrm{AlCl}}_{4}^{-}$ hardly diffuse. **Figure** **1**a depicts the mean‐square displacements (〈Δ*r*(*t*)^2^〉) of ions in Li_*x*_AlCl_3 + *x*_ at 375 K. The mobility of lithium is higher by more than an order of magnitude than chloride and aluminum ions. This indicates that the superionic conductivity in Li_*x*_AlCl_3 + *x*_ is governed mainly by lithium ions and that ${\mathrm{AlCl}}_{4}^{-}$ tetrahedra are responsible for sustaining the system in the solid state.

The diffusion of lithium ions is heterogeneous significantly. Figures 1b,c depict the self‐parts of van Hove correlation functions (*G*_*s*_(*r*, *t*)) and the non‐Gaussian parameters (α(*t*)) of lithium ions, respectively. If a lithium ion were to undergo Brownian diffusion, *G*_*s*_(*r*, *t*) should take the Gaussian form,^[^ ^60^ ^]^ i.e., $G_{s}\left(r,t\right)=\left(\frac{3}{2\pi \langle \Delta r{\left(t\right)}^{2}\rangle }\right)\exp\left(-\frac{3r^{2}}{2\langle \Delta r{\left(t\right)}^{2}\rangle }\right)$. As depicted in Figure 1b, however, $P_{{\mathrm{Li}}^{+}}\left(r,t=34\;\mathrm{ps}\right)=4\pi r^{2}G_{s}\left(r,t=34\;\mathrm{ps}\right)$ of lithium ions deviates significantly from being Gaussian and have two distinctive peaks around 0.5 and 4 Å. This indicates that lithium ions are categorized into two subsets: some lithium ions undergo hopping motions while others rattle. On the other hand, *G*_*s*_(*r*, *t*) of aluminum ions has a single peak around 0.5 Å, thus indicating that Al^3 +^ ions rattle within the tetrahedrons as expected for solids (Figure S2, Supporting Information). Figure 1f illustrates a representative trajectory of a lithium ion (a purple sphere) that undergoes a hopping motion. Grey tetrahedrons around the lithium ion are ${\mathrm{AlCl}}_{4}^{-}$ polyanions forming a cage for the lithium ion. While most lithium ions rattle within cages of polyanion tetrahedrons, some lithium ions hop, thus breaking free from the cage and diffusing.

The non‐Gaussian parameter (α(*t*)) provides a measure of how much and when lithium diffusion becomes heterogeneous. Figure 1c depicts α(*t*)'s of lithium ions for different compositions (*x*) at *T* = 375 *K*. α(*t*) reaches its maximum value (α_*max*_) at *t* = *t*_*max*_ ≈ 34 ps. α_*max*_ ranges from 1.3 to 2 depending on *x*, suggesting that the dynamic heterogeneity of lithium ions should be significant and comparable to other glass systems.

Both the lithium mobility and the dynamic heterogeneity are strongly dependent on the composition (*x*) of Li_*x*_AlCl_3 + *x*_. Figure 1d depicts the mobility *M* (defined as $M\equiv \langle \Delta r{\left(t=34\;\mathrm{ps}\right)}^{2}\rangle$) and the inverse of α_*max*_ of lithium ions as a function of *x*. Both *M* and 1/α_*max*_ show a non‐monotonic dependence on *x* with their maxima at *x* = 1. *M* depends significantly on *x* and *M* at *x* = 1 is about two times larger than at *x* = 0.25 and 3. In case of Na_2.25 − *x*_Y_0.25_Zr_0.75_Cl_6 − *x*_ electrolytes, the ionic conductivity at *x* = 1.625 is about two times larger than *x* = 1.375 and four times larger than *x* = 2.^[^ ^45^ ^]^ We also calculate the glass transition temperature (*T*_*g*_) of Li_*x*_AlCl_3 + *x*_ and find that *T*_*g*_ show an identical composition trend where *T*_*g*_ is the smallest at *x* = 1. This suggests that the dynamic heterogeneity should relate closely to the ion transport mechanism of IGSSEs.

On the other hand, the density (ρ) of Li_*x*_AlCl_3 + *x*_ increases monotonically with an increase in *x* (Figure 1e). Because the overall viscosity usually increases with an increase in ρ, the monotonic increase in ρ with *x* may not explain the non‐monotonic dependence of the lithium ion mobility on the composition. Therefore, the dynamic heterogeneity of lithium ions in Li_*x*_AlCl_3 + *x*_ should be a critical factor to understand the composition dependence.

### 2.2 The Transport Mechanism of Lithium Ions

**Figure 2:**
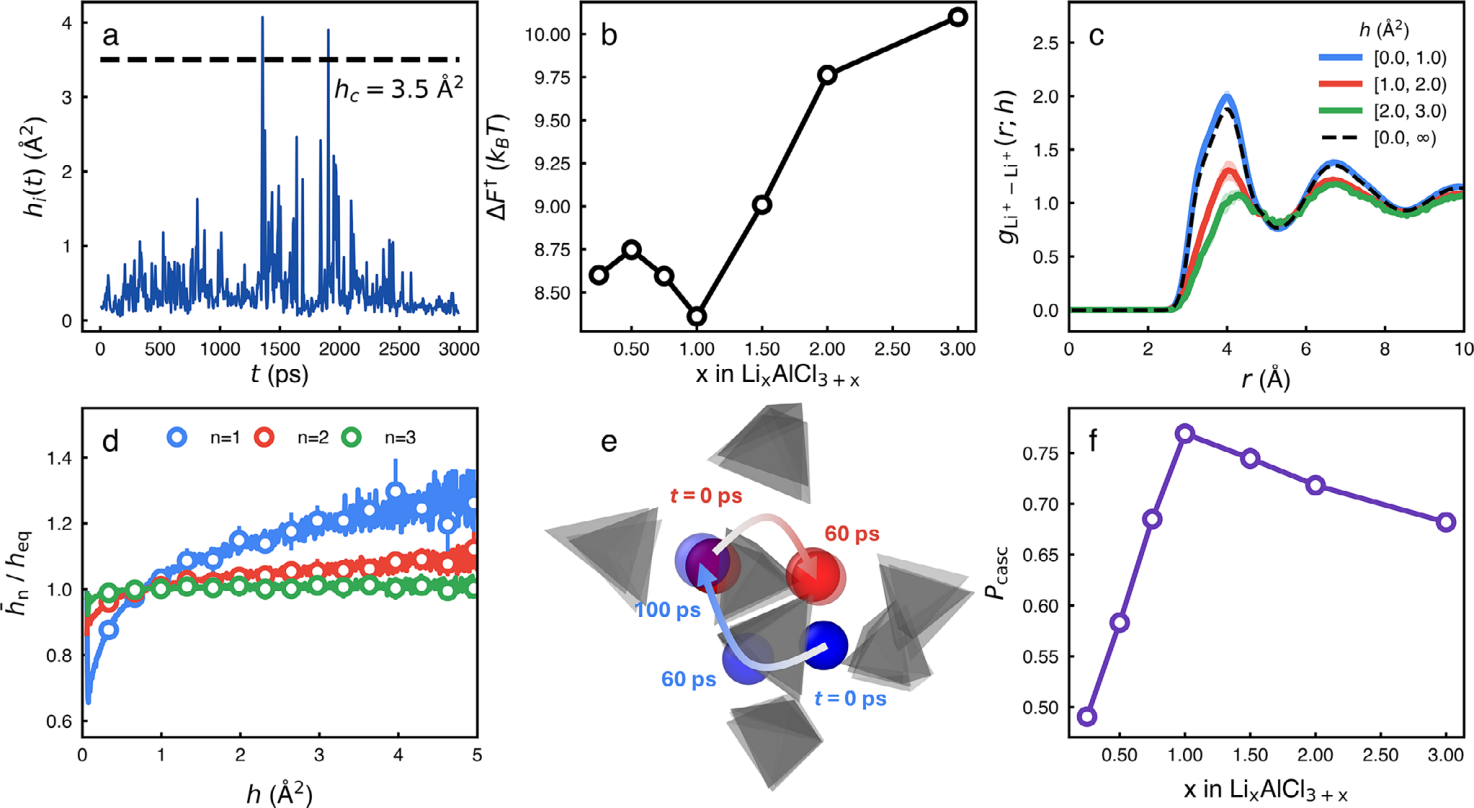
The hop function analysis and the cascading hopping of lithium ions. a) A representative hopping function (*h*_*i*_(*t*)) of a particular lithium ion. b) The free energy barrier (Δ*F*†) for a lithium ion to hop as a function of the composition *x*. c) The conditional radial distribution function ($g_{{\mathrm{Li}}^{+}-{\mathrm{Li}}^{+}}\left(r;h\right)$) between lithium ions. Here, one lithium ion hops with *h*_*i*_(*t*) = *h*. The dashed line corresponds to the overall radial distribution function $g_{{\mathrm{Li}}^{+}-{\mathrm{Li}}^{+}}\left(r\right)$ averaged over all values of *h*. d) ${\bar{h}}_{n}/h_{eq}$ as a function of *h*_*i*_(*t*) = *h* for different shells. ${\bar{h}}_{n}/h_{eq}$ indicates how much the lithium ion in the *n* ^th^ shell around a particular lithium ion (with *h*_*i*_(*t*) = *h*) is likely to hop. This quantifies how the hopping motions of neighbor lithium ions are correlated. e) The representative trajectory of two neighbor lithium ions undergoing a cascading hopping event. After the red lithium ion hops out of the cage of ${\mathrm{AlCl}}_{4}^{-}$ polyanions, the blue lithium ion hops into the cage. f) The fraction (*P*_casc_) of the cascading hopping motion out of total number of hopping motions as a function of the composition (*x*).

Hop function analysis has been employed to investigate the transport mechanism of dynamically heterogeneous systems.^[^ ^61^, ^62^, ^63^, ^64^ ^]^ **Figure** **2**a depicts a temporal profile of the hop function (*h*_*i*_(*t*)) of a certain *i* ^th^ lithium ion at *T* = 375 *K* in LiAlCl_4_. *h*_*i*_(*t*) is sensitive to the translational motion of the lithium ion such that *h*_*i*_(*t*) fluctuates significantly. Because we decide that a lithium ion hops only when *h*_*i*_(*t*) ⩾ *h*_*c*_ = 3.5Å ^2^, the lithium ion in Figure 2a undergoes hopping motions two times during 3 ns.

The free energy barrier (Δ*F*†) required for a lithium ion to overcome to hop beyond the threshold value *h*_*c*_ is also a non‐monotonic function of the composition *x* with its the minimum at *x* = 1 (Figure 2b). When the mobility *M* is the highest (*x* = 1), the free energy barrier for the hopping motions is the minimum with Δ*F*† ≈ 8.3 *k*_*B*_ *T* while Δ*F*† increases up to 10 *k*_*B*_ *T* at *x* = 3. When *x* = 1, lithium ions may undergo a hopping motion with the smallest free energy barrier such that the ion mobility can be highest. This suggests that the hop function (*h*_*i*_(*t*)) should be a proper reaction coordinate to explain the composition dependency of the ion mobility in Li_*x*_AlCl_3 + *x*_.

In order to elucidate the transport mechanism of lithium ions in Li_*x*_AlCl_3 + *x*_, we investigate the structural correlation that facilitates the hopping motion of lithium ions. We calculate the conditional radial distribution function ($g_{{\mathrm{Li}}^{+}-{\mathrm{Li}}^{+}}\left(r;h\right)$) of the lithium ion that undergoes the hopping motion with *h*_*i*_(*t*) = *h*. $g_{{\mathrm{Li}}^{+}-{\mathrm{Li}}^{+}}\left(r;h\right)$ allows us to investigate how neighbor lithium ions are correlated spatially with the lithium ion with *h*_*i*_(*t*) = *h*. Interestingly, $g_{{\mathrm{Li}}^{+}-{\mathrm{Li}}^{+}}\left(r;h\right)$ depends strongly on the value of *h* (Figure 2c). When 0 ⩽ *h* < 1 Å ^2^ and lithium ions hardly hop but only rattle, $g_{{\mathrm{Li}}^{+}-{\mathrm{Li}}^{+}}\left(r;h\right)$ is almost identical to the overall radial distribution function $g_{{\mathrm{Li}}^{+}-{\mathrm{Li}}^{+}}\left(r\right)$ (dashed line) and shows a high peak at *r* ≈ 4 Å. This is consistent with our observation of $P_{{\mathrm{Li}}^{+}}\left(r,t=34\;\mathrm{ps}\right)$, where most lithium ions only rattle (Figure 1b). On the other hand, as lithium ions hop with a large value of *h* ⩾ *h*_*c*_, the first peak of $g_{{\mathrm{Li}}^{+}-{\mathrm{Li}}^{+}}\left(r;h\right)$ diminishes significantly. This suggests that in order for a lithium ion to hop and diffuse, neighbor lithium ions (especially in the first shell) should be depleted with vacancies of neighbor lithium ions.

We find that the depletion of neighbor lithium ions facilitates the cooperative motions of those neighbor lithium ions, i.e., the cascading hopping event of lithium ions. We estimate how the hopping motion of the neighbor *j* ^th^ lithium ion is affected by the hopping motion of the *i* ^th^ lithium ion of interest. We categorize neighbor lithium ions into different shells based on the radial distribution function ($g_{{\mathrm{Li}}^{+}-{\mathrm{Li}}^{+}}\left(r\right)$). For neighbor *j* ^th^ lithium ions in the *n* ^th^ shell around the *i* ^th^ lithium ion, we calculate the average (${\bar{h}}_{n}$) of the hop function *h*_*j*_(*t*). And we also calculate the ensemble average (*h*_*eq*_) of *h*_*j*_(*t*) for all shells, which corresponds to the average of *h*_*i*_(*t*) of all lithium ions.

Figure 2d depicts ${\bar{h}}_{n}/h_{eq}$ as a function of *h*_*i*_(*t*) for different shells. In case of lithium ions in the first shell, ${\bar{h}}_{1}/h_{eq}\;>1$ especially when *h*_*i*_(*t*) ⩾ *h*_*c*_. This indicates that when the *i* ^th^ lithium ion of interest hops, Li^+^ ions in the first shell are also likely to undergo hopping motions. On the other hand, in case of lithium ions in outer shells (*n* ⩾ 2), ${\bar{h}}_{n}/h_{eq}\;\approx 1$, thus suggesting that the hopping motions of lithium ions in the outer shells are hardly affected by the hopping motion of the *i* ^th^ lithium ion.

Figure 2e shows a representative trajectory for the cascading hopping event. Here, blue and red lithium ions undergo cooperative hopping motions. The red lithium ion hops out of a cage of ${\mathrm{AlCl}}_{4}^{-}$ polyhedrons and then the blue lithium ion hops in to fill the vacancy generated by the red lithium ion. This cascading event of hopping motions should be the main transport mechanism of Li_*x*_AlCl_3 + *x*_.

In order for the cascading hopping motions of lithium ions to occur effectively, both neighbor vacancies and neighbor lithium ions should be present. When *x* is small and the lithium ion concentration is low, there are many vacancies but the system lacks a sufficient number of neighbor lithium ions. On the other hand, when *x* is large with high lithium ion concentration, there are many neighbor lithium ions but the system does not have a sufficient amount of neighbor vacancies for the cascading event. Therefore, there exists an optimal composition *x* where both neighbor vacancies and neighbor lithium ions are sufficient for the cascading event. In Li_*x*_AlCl_3 + *x*_, *x* = 1 corresponds to the optimal composition.

We calculate the fraction (*P*_casc_) of the cascading hopping motion out of total number of hopping motions to corroborate the above argument (Figure 2f). We consider a hopping motion as a cascading hop if the hopping motion of the *i* ^th^ lithium ion occurs with *h*_*i*_(*t*) > *h*_*c*_ within 10 ps after the *j* ^th^ neighbor lithium ion (within the 1^st^ shell of the *i* ^th^ lithium ion) undergoes hopping motion with *h*_*j*_(*t*) > *h*_*c*_. Then, *P*_casc_ is the ratio of the number of cascading hopping motions to the total number of lithium ion hopping motions. *P*_*casc*_ is quite large up to about 0.76, thus indicating that the cascading hopping event should be the main transport mechanism. More interesting is that *P*_casc_ depends on the composition (*x*) as in the lithium ion mobility (*M*) and the free energy barrier (Δ*F*†). This indicates that when *x* = 1, the cascading event should be most efficient such that the ion mobility should show such a non‐monotonic composition dependence.

### 2.3 The Correlation Between Lithium Hopping and ${\mathrm{AlCl}}_{4}^{-}$ Rotation

**Figure 3:**
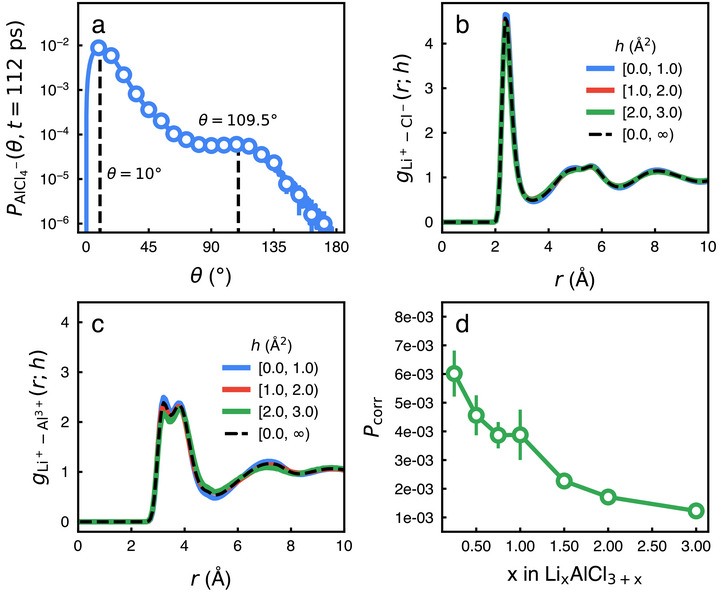
The weak coupling between the diffusion of a lithium ion and the rotation of its neighbor polyanions. a) The self‐part of the rotational van Hove correlation function (*P*(θ, *t* = 112 ps)) of a polyanion (${\mathrm{AlCl}}_{4}^{-}$) at *t* = 112 ps and *x* = 1, indicating that the polyanion rotation occurs but is also heterogeneous. b) The conditional radial distribution function ($g_{{\mathrm{Li}}^{+}-{\mathrm{Cl}}^{-}}\left(r;h\right)$) between a chloride ion and the lithium ion that undergoes the hopping motion with *h*_*i*_(*t*) = *h*, quantifying how much neighbor chloride ion would correlate spatially with a lithium ion that hops. The dashed line corresponds to the overall radial distribution function $g_{{\mathrm{Li}}^{+}-{\mathrm{Cl}}^{-}}\left(r\right)$ averaged over all values of *h*. c) The conditional radial distribution function ($g_{{\mathrm{Li}}^{+}-{\mathrm{Al}}^{3+}}\left(r;h\right)$) between an aluminium ion and the lithium ion that undergoes the hopping motion with *h*_*i*_(*t*) = *h*, quantifying how much neighbor aluminium ion would correlate spatially with a lithium ion that hops. The dashed line corresponds to the overall radial distribution function $g_{{\mathrm{Li}}^{+}-{\mathrm{Al}}^{3+}}\left(r\right)$ averaged over all values of *h*. d) The fraction (*P*_corr_) of correlated hopping motions, i.e. the fraction of lithium ion hopping events that occur within 10 ps after the neighbor ${\mathrm{AlCl}}_{4}^{-}$ polyanions rotates by more than 90°. *P*_corr_ is very small and a monotonically decreasing function of the composition *x*, thus indicating that the paddlewheel effect would not be the dominant ion transport mechanism.

The correlation between neighbor polyanions and lithium ion hopping motions is weak unlike what has been expected from the paddlewheel mechanism. We find that ${\mathrm{AlCl}}_{4}^{-}$ polyhedrons rotate even at 375 K to some extent. **Figure** **3**a depicts the self‐part of the rotational van Hove correlation function (*P*(θ, *t* = 112 ps)) of a polyanion (${\mathrm{AlCl}}_{4}^{-}$) at *t* = 112 ps and *x* = 1. *P*(θ, *t* = 112 ps) shows how much the chemical bond between aluminum and chloride ions rotates during 112 ps. The presence of two distinct peaks (θ ≈ 10° and 109.5°) indicates that the rotation of ${\mathrm{AlCl}}_{4}^{-}$ should be quite heterogeneous: most polyanion tetrahedrons rattle rotationally within θ ≈ 10° but a few polyanion tetrahedrons rotate by θ = 109.5°. The rotation of the tetrahedron by 109.5° is dominant due to its tetrahedral symmetry, i.e., the rotation by θ = 109.5° leads to symmetrically degenerate configurations of the polyanion.

The rotation of ${\mathrm{AlCl}}_{4}^{-}$ polyhedrons hardly correlates with the translational hopping motion of lithium ions. We calculate the conditional radial distribution functions ($g_{{\mathrm{Li}}^{+}-{\mathrm{Cl}}^{-}}\left(r;h\right)$ and $g_{{\mathrm{Li}}^{+}-{\mathrm{Al}}^{3+}}\left(r;h\right)$) between ionic species of polyanions (either Cl^−^ or Al^3 +^) and the lithium ion that undergoes the hopping motion with *h*_*i*_(*t*) = *h*. This allows us to evaluate how neighbor polyanions would correlate spatially with a lithium ion that hops. Figure 3b depicts both $g_{{\mathrm{Li}}^{+}-{\mathrm{Cl}}^{-}}\left(r;h\right)$ (solid) and $g_{{\mathrm{Li}}^{+}-{\mathrm{Cl}}^{-}}\left(r\right)$ (dashed). Note that $g_{{\mathrm{Li}}^{+}-{\mathrm{Cl}}^{-}}\left(r\right)$ is the overall radial distribution function between lithium and chloride ions regardless of the hopping motion of lithium ions. There is hardly a difference between $g_{{\mathrm{Li}}^{+}-{\mathrm{Cl}}^{-}}\left(r;h\right)$ and $g_{{\mathrm{Li}}^{+}-{\mathrm{Cl}}^{-}}\left(r\right)$. And $g_{{\mathrm{Li}}^{+}-{\mathrm{Cl}}^{-}}\left(r;h\right)$ is hardly dependent on the value of *h*, too. Similarly, $g_{{\mathrm{Li}}^{+}-{\mathrm{Al}}^{3+}}\left(r;h\right)$’s are hardly dependent on the value of *h* and is almost identical to the overall radial distribution function $g_{{\mathrm{Li}}^{+}-{\mathrm{Al}}^{3+}}\left(r\right)$ (dashed line) (Figure 3c). This shows clearly that the spatial arrangement of polyanions around a lithium ion should be independent of whether the lithium ion hops or not.

In order to quantify how much the ${\mathrm{AlCl}}_{4}^{-}$ rotation affects the lithium hopping, we calculate the probability (*P*_corr_) of correlated hopping motions. We decide that when θ ⩾ 90°, the ${\mathrm{AlCl}}_{4}^{-}$ polyhedron rotates. θ = 90° divides *P*(θ, *t* = 112 ps) into i) polyhedrons that only rattle rotationally and ii) polyhedrons that undergo rotational hopping motions. Then, we search for neighbor ${\mathrm{AlCl}}_{4}^{-}$ polyhedrons around a lithium ion by calculating the distance ($r_{{\mathrm{Li}}^{+}-{\mathrm{Al}}^{3+}}$) between the lithium ion and aluminum ion. We decide that ${\mathrm{AlCl}}_{4}^{-}$ polyhedrons are neighbor polyanions when $r_{{\mathrm{Li}}^{+}-{\mathrm{Al}}^{3+}}\leq 5.1\;Å$, because $r_{{\mathrm{Li}}^{+}-{\mathrm{Al}}^{3+}}\leq 5.1\;Å$ corresponds to the first shell of $g_{{\mathrm{Li}}^{+}-{\mathrm{Al}}^{3+}}\left(r\right)$. If one of neighbor polyanions rotate and then the lithium ion undergo hopping motion (with *h*_*i*_(*t*) ⩾ *h*_*c*_) within 10 ps after the rotation, we consider the hopping motion as a correlated hopping motion. We calculate the fraction (*P*_corr_) of the correlated hopping motion out of total number of hopping motions during our simulations. *P*_corr_ allows us to evaluate how much lithium ion hopping motions occur with or without polyanion rotations.

*P*_corr_ is very small (Figure 3d), thus indicating that most lithium hopping motions occur regardless of whether the neighbor ${\mathrm{AlCl}}_{4}^{-}$ polyanions rotate or not. *P*_corr_ decreases from 0.006 to below 0.002 as *x* in Li_*x*_AlCl_3 + *x*_ increases. Less than 1% of hopping motions may correlate with polyanion rotation. More significantly, *P*_corr_ shows a monotonic dependence on the composition, which cannot explain the non‐monotonic dependence of the mobility on the composition (*x*).

### 2.4 The Cascading Hopping Motions of Li Ions in Amorphous LPS

**Figure 4:**
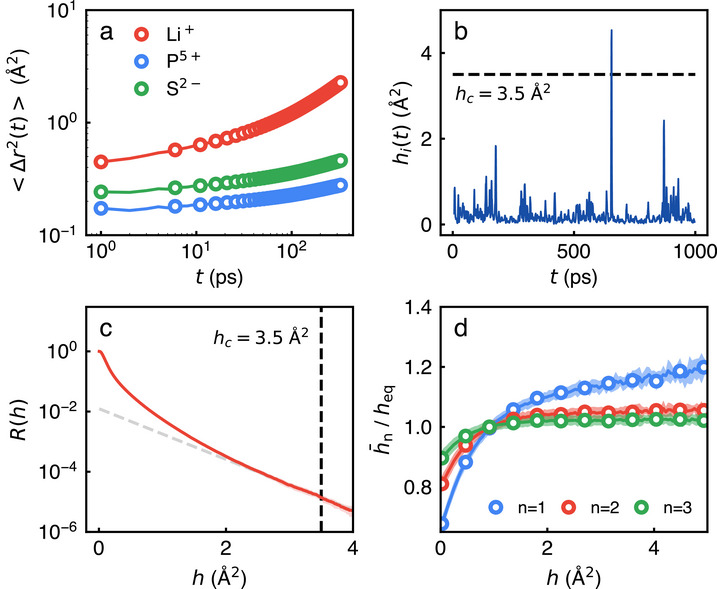
Dynamic properties and hop function analyses of amorphous LPS obtained from MLMD simulations. a) The mean‐square displacements (〈Δ*r*(*t*)^2^〉's) of ions, b) the hop function (*h*_*i*_(*t*)) of a certain *i*th Li ion, c) the cumulative probability (*R*(*h*)), and d) $\bar{h_{n}}/h_{eq}$ of Li ions in LPS.

Our approach to explore the ion conduction mechanism along with MLMD simulations and the hop function analysis should be quite general and can be extended easily to various types of heterogeneous systems and SSEs. We also construct MLP for amorphous LPS (75Li_2_S·25P_2_S_5_) by following the same procedure employed for Li_*x*_AlCl_3 + *x*_. We carry out MLMD simulations at T = 300 K and density of 1.882 ± 0.002 *gcm* ^−^3, and hop function analysis. We find that Li ions are dispersed well while P and S ions construct tetrahedrons. Our MLMD simulation results for $g_{{\mathrm{Li}}^{+}-{\mathrm{Li}}^{+}}\left(r\right)$ are also consistent with previous studies.^[^ ^29^, ^30^, ^57^ ^]^ More details and simulation results for structural properties are provided in Supporting Information. As shown in **Figure** **4** a for the mean‐squared displacement (〈Δ*r*(*t*)^2^〉), Li ions are relative mobile and responsible for ion conduction while tetrahedral polyanions are hardly diffusive and responsible for sustaining the system in solid state. The hop function analysis for Li ions suggests that Li ions in amorphous LPS also carry out infrequent hopping motions (Figure 4b). By calculating the cumulative probability (*R*(*h*)), we find that *h*_*c*_ ≈ 3.5Å in case of amorphous LPS. Interestingly, we find from the hop function analysis that $\bar{h_{1}}/h_{eq}\;>1$ when *h*_*i*_(*t*) > *h*_*c*_ (Figure 4d). This indicates that when the *i*th Li ion in amorphous LPS undergoes a hopping motion, other Li ions in its first shell are also likely to undergo hopping motions. This suggests that even for amorphous LPS, the cascading hopping motion would be the main transport mechanism. In the future study, we plan to extend our approach to investigate the transport mechanisms of both amorphous and crystalline LPS systems.

## 3 Discussion

We perform large‐scale MLMD simulations using MLP and the hop function analysis to investigate the ion transport mechanism of a model IGSSE of Li_*x*_AlCl_3 + *x*_. The potential energy and force of the MLP are accurate compared to DFT calculations and are successful at describing the amorphous structure of Li_*x*_AlCl_3 + *x*_: while lithium ions are dispersed well, ${\mathrm{AlCl}}_{4}^{-}$ polyanions form stable tetrahedra during our simulations without a long‐range order.

The diffusion and mobility of lithium ions are heterogeneous significantly and non‐monotonically dependent on the composition (*x*) of Li_*x*_AlCl_3 + *x*_. Some lithium ions undergo hopping motions (which are responsible for the high conductivity of IGSSEs) while other lithium ions and ${\mathrm{AlCl}}_{4}^{-}$ polyanions hardly diffuse during nanoseconds (which are responsible for sustaining IGSSEs in the solid state). Such dynamic heterogeneity of lithium ions are characterized by the non‐Gaussian parameter (α(*t*)) and its maximum value α_*max*_. We find that the mobility *M* of lithium ions shows the same non‐monotonic composition‐dependency with 1/α_*max*_, thus indicating that the dynamic heterogeneity should be a key concept to explain the composition dependence of the ion conductivity of IGSSEs.

The hop function analysis allows us to investigate how the local environment of a lithium ion would correlate with its hopping motion. We successfully identify the hopping motions of lithium ions from the hop function analysis and also estimate the free energy barrier (Δ*F*†) required for the lithium ions to overcome to hop. Δ*F*† also shows the identical non‐monotonic composition dependence, which suggests that the hop function (*h*_*i*_(*t*)) would be a proper reaction coordinate to describe the complex mobility of lithium ions. More analyses based on the hop function (such as $g_{{\mathrm{Li}}^{+}-{\mathrm{Li}}^{+}}\left(r;h\right)$ and ${\bar{h}}_{n}/h_{eq}$) illustrate that a lithium ion hops to diffuse when its neighbor lithium ions are depleted, thus causing the cascading hopping of lithium ions. We believe that the cascading hopping motions of lithium ions should be a main ion transport mechanism. The fraction (*P*_casc_) of the cascading hopping motion reaches up to 0.75, indicating that most hopping motions accompany the hopping motions of neighbor lithium ions.

We also find that in case of Li_*x*_AlCl_3 + *x*_, the rotation of ${\mathrm{AlCl}}_{4}^{-}$ polyanions hardly affects the lithium ion transport. The hop function analysis and $g_{{\mathrm{Li}}^{+}-{\mathrm{Cl}}^{-}}\left(r;h\right)$ show that the hopping motion of a lithium ion hardly correlates with the local arrangement of polyanions. And the probability (*P*_corr_) of a lithium ion hopping during 10 ps after the neighbor ${\mathrm{AlCl}}_{4}^{-}$ polyanions rotate is very small below 0.006 and decreases monotonically with *x*. This indicates that the paddlewheel effect would not be able to explain the composition dependence of the ion conductivity of IGSSEs.

The correlation between the hopping motion of a Li ion and its local environment plays a critical role in facilitating the ion conduction of SSEs. The correlation with the rotation of neighbor polyanions is considered in the paddlewheel mechanism,^[^ ^19^, ^20^, ^21^ ^]^ whereas the correlation with neighbor Li ions is considered as a critical component in the concerted migration mechanism.^[^ ^25^, ^26^, ^27^, ^28^ ^]^ There has been an intense debate over the presence of paddlewheel effect^[^ ^32^, ^33^ ^]^ and recent AIMD simulations reported that the rotation of some polyanions could affect the Li ion diffusion even in a negative way.^[^ ^23^, ^24^ ^]^ Our simulation results for Li_*x*_AlCl_3 + *x*_ also show the weak correlation between the polyanion rotation and the Li ion hopping.

AIMD simulation studies for various crystalline systems (Li_7_La_3_Zr_2_O_12_, Li_10_GeP_2_S_12_, and Li_1.3_Al_0.3_Ti_1.7_(PO_4_)_3_) showed that the concerted migration with a strong correlation between Li ion hopping motions should be the main transport mechanism.^[^ ^25^, ^26^, ^27^, ^28^ ^]^ The concerted migration mechanism is similar to the cascading hopping motions in the sense that the hopping motions of neighbor Li ions are strongly correlated and the hopping motion of a certain Li ion facilitates the hopping motion of its neighbor Li ions. In case of crystalline systems, the ion migration channels though high and low energy sites could be defined readily, and the activation energy for the ion hopping were relatively well‐defined. In case of amorphous systems, however, the ion migration channels and ion sites are poorly defined due to the absence of well‐defined lattice sites, and the activation energy for the ion migration should be heterogeneous due to the very rugged free energy surface of amorphous solids (often with deep local minima). This is why the hop function analysis can make a contribution to the identification of the transport mechanism of amorphous solids. More importantly, the hop function analysis and the cascading hopping motions reveal that the non‐monotonic composition dependence of the ion conductivity originates from a subtle balance between the concentrations of local lithium ions and the lithium vacancies (Figure 2), which should be an important guiding rule when optimizing amorphous solid electrolytes.

## 4 Experimental Section

### The Construction of The Machine‐Learning Potential for Li_*x*_AlCl_3 + *x*_

The machine learning potential was constructed by using AIMD simulation results as training data. In case of AIMD simulations, the initial configurations of small scale LiAlCl_4_ was prepared by placing 16 lithium, 16 aluminum, and 64 chloride ions at random positions at three different densities (ρ's) of ρ ≈ 0.995, 1.99, and 2.99 *g*/*cm* ^3^. AIMD simulations under isothermal and isochoric conditions were performed by employing Vienna Ab initio Simulation Package (VASP)^[^ ^65^, ^66^ ^]^ with a Nosé‐Hoover thermostat at 1000 K and a 2 fs time step. The Perdew‐Burke‐Ernzerhof (PBE) generalized gradient approximation exchange‐correlation functional^[^ ^67^ ^]^ and projector augmented wave potentials^[^ ^68^ ^]^ (PAW_PBE Li_sv, PAW_PBE Al, and PAW_PBE Cl) were employed. A plane wave energy cutoff of 520 eV and a Γ‐centered 1 × 1 × 1 *k*‐point mesh were applied. Post‐equilibrium configurations were selected at 20 fs intervals for high‐level DFT calculations using the optB88‐vdW exchange‐correlation functional with PBE correlation^[^ ^69^, ^70^ ^]^ with other settings unchanged. A total of 820 configurations were obtained at three different densities. The configurations were divided into subsets: 90% for training MLP and 10% for testing the MLP. The details of density functional calculations and AIMD simulations are provided in Supporting Information.

Moment tensor potential (MTP)^[^ ^71^ ^]^ was chosen to describe the interatomic potential. The Machine Learning of Interatomic Potentials (MLIP) software package^[^ ^72^, ^73^, ^74^ ^]^ was used to train MTP and integrate it into Large‐scale Atomic/Molecular Massively Parallel Simulator (LAMMPS).^[^ ^75^ ^]^ The maximum level of the basis function of MTP and the cutoff distance were set to 16 and 5 Å, respectively. The weights on errors in energy, force, and stress (*w*_*e*_, *w*_*f*_, and *w*_*s*_) were assigned as 1, 0.01, and 0.001, respectively. A learning‐on‐the‐fly molecular dynamics (LOFT‐MD) was employed for efficient MTP training with extrapolation boundaries (γ_select_, γ_break_) = (2, 10). Configurations acquired during simulation with extrapolation grade (γ) within these boundaries were saved and added to the dataset after DFT calculations. If a configuration exceeds the γ_break_ threshold, this configuration was considered untrustworthy and the simulation terminates. A stable MTP is defined as one that completes a 1 ns simulation with a 1 fs time step without generating any configurations that require DFT calculations. LOFT‐MD was initially performed on a 96 atom LiAlCl_4_ system under isothermal‐isobaric conditions at 1 bar. More details for DFT calculations and MTP potential are provided in the Supporting Information.

The potential energy and the force of the MLP were in an excellent agreement with DFT calculations (Figure S3, Supporting Information). The mean absolute error (MAE) and the root‐mean squared error (RMSE) of the potential energies of MLP are 0.004 and 0.005 eV/atom, respectively. In case of interatomic forces, MAE and RMSE are 0.138 and 0.186 eVÅ^−1^, respectively. A benchmark test was carried out for a small system of 96 atoms at 1000 K and find that the machine learning molecular dynamics (MLMD) with MLP was faster by five orders of magnitude than AIMD. The RMSE values of the error in the forces are relatively large compared to those for crystalline systems^[^ ^28^, ^76^ ^]^ but were comparable to those of other solid electrolytes.^[^ ^57^, ^77^ ^]^ In order to ensure that the force errors did not propagate into the inaccuracies in physical properties, the radial distribution functions and the angle distribution functions of polyanions obtained from AIMD and MLMD simulations were compared, and find that they are almost identical to each other (Figures S5 and S6, Supporting Information).

### The Large‐Scale Machine Learning Molecular Dynamics (MLMD) Simulations

The initial configurations of large‐scale Li_*x*_AlCl_3 + *x*_ were constructed by placing ions at random positions. The initial configurations were equilibrated and melted at high temperature of 1000 K by performing MLMD simulations under isothermal‐isobaric conditions at 1 bar during 500 ps. The potential energy was verified to converge well during the equilibration simulations. Then, the systems were cooled from 1000 to 100 K at a cooling rate of 450 K/ns at 1 bar. The glass transition temperature, *T*_*g*_ was determined by analyzing the gradient change in the temperature‐density curve. With the configuration at 375 K obtained from cooling process, additional equilibration isotherml‐isobaric MD simulations were performed at 1 bar during 2 ns. Then, the production run was carried out at isothermal‐isochoric condition. A time step of 1 fs was used for all simulations.

To investigate the ion diffusion, the mean squared displacements of the ions (〈Δ*r*(*t*)^2^〉) i.e., $\left\langle \Delta r{\left(t\right)}^{2}\right\rangle =\left\langle {\left(\vec{r}\left(t\right)-\vec{r}\left(0\right)\right)}^{2}\right\rangle$ was calculated. Here, $\vec{r}\left(t\right)$ is the position vector of the ion at time *t* and 〈⋅⋅⋅〉 denotes an ensemble average. The self‐part of van Hove correlation function $G_{s}\left(r,t\right)=\left\langle \delta (r-\right|{\vec{r}}_{i}\left(t\right)-{\vec{r}}_{i}\left(0\right)|)\rangle$ was calculated. *G*_*s*_(*r*, *t*) measures how much an ion would diffuse during time *t*. The non‐Gaussian parameter, $\alpha \left(t\right)=\frac{3\langle \Delta r{\left(t\right)}^{4}\rangle }{5{\left\langle \Delta r{\left(t\right)}^{2}\right\rangle }^{2}}-1$, quantifies how much the ion diffusion deviates from the Brownian dynamics, i.e., the extent of the dynamic heterogeneity. The self‐part of the rotational van Hove correlation function (*P*(θ, *t*) = 〈δ(θ − |θ_*i*_(*t*) − θ_*i*_(0)|)〉) of a polyanion (${\mathrm{AlCl}}_{4}^{-}$) was calculated to investigate the rotation of ${\mathrm{AlCl}}_{4}^{-}$. A normalized vector from aluminum atom to one of chloride atoms was defined (Figure S1b, Supporting Information). Here, θ(*t*) is the angle by which the normalized vector rotates during time *t*.

### The Hop Function Analysis

The hopping motions of lithium ions was investigated by employing a hop function, *h*_*i*_(*t*), defined as

(1) $$h_{i}\left(t\right)\equiv \sqrt{{\left\langle {\left({\vec{r}}_{i}\left(t\right)-{\left\langle {\vec{r}}_{i}\left(t\right)\right\rangle }_{B}\right)}^{2}\right\rangle }_{A}{\left\langle {\left({\vec{r}}_{i}\left(t\right)-{\left\langle {\vec{r}}_{i}\left(t\right)\right\rangle }_{A}\right)}^{2}\right\rangle }_{B}}$$

where ${\vec{r}}_{i}\left(t\right)$ denotes the position vector of the *i*th lithium ion at time *t*. 〈*X*〉_*A*_ and 〈*X*〉_*B*_ are the time averages of *X* in two different time windows *A* and *B*, i.e., ${\left\langle X\right\rangle }_{A}=\frac{1}{\Delta t/2}\int_{t-\Delta t/2}^{t}X\left(\tau \right)d\tau$ and ${\left\langle X\right\rangle }_{B}=\frac{1}{\Delta t/2}\int_{t}^{t+\Delta t/2}X\left(\tau \right)d\tau$. *h*_*i*_(*t*) responds sensitively when a particle undergoes a hopping motion. *h*_*i*_(*t*) was, therefore, employed successfully to elucidate the transport mechanism of dynamically heterogeneous systems including supercooled water and dense granular media.^[^ ^61^, ^62^, ^64^ ^]^ In this study, Δ*t* = 1 ps was employed.

In order to distinguish the hopping motion from rattling motions of lithium ions, the distribution function *P*(*h*) of the values of *h*_*i*_(*t*) and its cumulative distribution $R\left(h\right)\equiv \int_{h}^{\infty }P\left(h^{\prime }\right)dh^{\prime }$ were calculated (Figure S4, Supporting Information). *R*(*h*) decreases quickly up to *h* = h* and exhibits an exponential decay for *h* ⩾ h* with less frequent but large hopping motions. Small rattling motions with *h* < h* were correlated with each other while large hopping motions with *h* ⩾ h* were independent events such that *R*(*h*) decays exponentially at *h* ⩾ h*. h* was, then, a threshold value between the hopping and the rattling motions. The value of h* was determined by using the equation ${\left(\frac{d\ln R(h)}{dh}\right)}_{h=h^{*}}=0$ for the Li_*x*_AlCl_3 + *x*_. h* depends on the composition of Li_*x*_AlCl_3 + *x*_, ranging from 1.8 to 3 Å ^2^. Therefore, a sufficiently large value of *h* = *h*_*c*_ = 3.5 Å ^2^ as a criterion was chosen to determine whether a lithium ion carries out an independent hopping motion or not.

The free energy profile (*F*(*h*)) was calculated for the hopping motions of lithium ions by using the relation *F*(*h*) = −*k*_*B*_ *T*ln *P*(*h*).^[^ ^64^ ^]^ This allowed to estimate the free energy barrier (Δ*F*†) for the hopping motion defined as Δ*F*† ≡ *F*(*h*_*c*_) − *F*_*min*_, where *F*_*min*_ denotes the minimum value of *F*(*h*). The systematically how the local environment of a lithium ion would correlate with its hopping motion can also be investigated. The conditional radial distribution function ($g_{{\mathrm{Li}}^{+}-A}\left(r;h\right)$) was calculated between the ion *A* of the local environment and the lithium ion that undergoes the hopping motion with *h*_*i*_(*t*) = *h*, i.e.

(2) $$g_{{\mathrm{Li}}^{+}-A}\left(r;h\right)=\frac{V}{4\pi r^{2}N_{{\mathrm{Li}}^{+}}^{\prime }N_{A}}\sum_{h_{i}=h}\sum_{j}^{N_{A}}\left\langle \delta (r-\right|{\vec{r}}_{i}-{\vec{r}}_{j}\left|)\right\rangle$$

where *V* and *N*_A_ are the system volume, and the number of *A* ions, respectively. $N_{{\mathrm{Li}}^{+}}^{\prime }$ is the number of the lithium ions with *h*_*i*_(*t*) = *h*. ${\vec{r}}_{i}$ denotes the position vectors of the *i* ^th^ atom.

## Conflict of Interest

The authors declare no conflict of interest.

## Author Contributions

B. K. and J. Y. contributed equally to this work. These authors constructed the MLP, carried out MLMD simulations, and analyzed the data. S. S., J. J., and B. J. S. conceived the strategies of combining the hop function analysis and MLMD simulations for IGSSEs. S. S., J. J., and B. J. S. also supervised the project. All authors discussed the results and implications and commented on the manuscript at all stages.

## Supporting information

Supporting Information

## Data Availability

The data that support the findings of this study are available from the corresponding author upon reasonable request.
