# Supplementary material for: Non‐Monotonic Ion Conductivity in Lithium‐Aluminum‐Chloride Glass Solid‐State Electrolytes Explained by Cascading Hopping
Source: Adv Sci (Weinh). 2025 Sep 17;12(45):e09205. doi: 10.1002/advs.202509205 (PMC12677655; doi:10.1002/advs.202509205)
Supplement: Supplementary file 1 — Supporting Information [file ADVS-12-e09205-s001.pdf]

Supporting Information for

Cascading Hopping as Ion Conduction Mechanism of Inorganic Glass Solid-State Electrolytes of Lithium-Aluminum-Chloride with Non-monotonic Composition Dependence

Beomgyu Kang[1†], Jina Yu[1†], Shinji Saito[4,5*], Jihyun Jang[1,3*] and Bong June Sung[1,2*]

[1]*Department of Chemistry, Sogang University, Seoul 04107, Republic of Korea,*
[2]*Institute of Biological Interfaces, Sogang University, Seoul 04107, Republic of Korea,*
[3]*Center for Nano Materials, Sogang University, Seoul 04107, Republic of Korea,*
[4]*Institute for Molecular Science, Myodaiji, Okazaki, Aichi 444-8585, Japan,*
[5]*The Graduate University for Advanced Studies (SOKENDAI), Myodaiji, Okazaki, Aichi, 444-8585, Japan*

Supplementary Figures

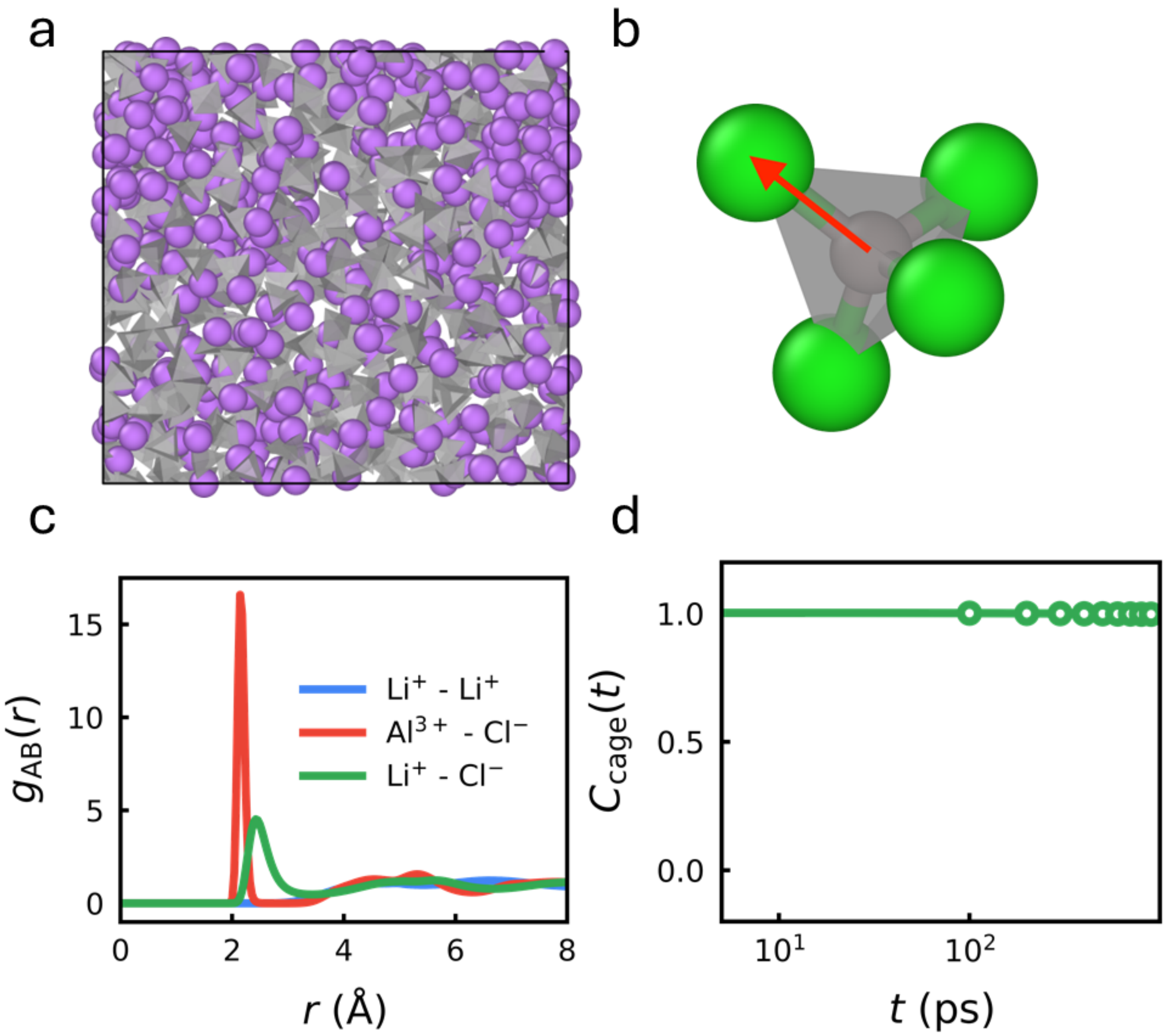

Supplementary Figure S1. Structural features of amorphous $LiAlCl_4$ at 375 K: a. The representative snapshot of $LiAlCl_4$ melts at 375 K where purple spheres and grey polyhedra indicate lithium ions and $AlCl_4^-$ polyanions, respectively. **b.** A representative snapshot of the tetrahedral structure of $AlCl_4^-$ polyanion. The red vector, which is from the central aluminum ion to neighboring chloride ion, depicts the characteristic vector for identifying polyanion rotation. This red vector is utilized to calculate cage correlation, $C_{\mathrm{cage}}(t)$ (Equation S2.), and the rotational van Hove correlation function of a polyanion, $P(\theta, t)$. **c.** The radial distribution functions, $g_{AB}(r)$'s, of ions in $LiAlCl_4$ at 375 K. The strong correlation between aluminum and chloride ions can be identified. No other significant long-range order between ions can be found. **d.** The cage correlation function, $C_{\mathrm{cage}}(t)$ quantifies how stable a polyanion tetrahedron of $AlCl_4^-$ would be. More details on $C_{cage}(t)$ is provided in Supplementary Equation S2. $C_{cage}(t)$ remains close to 1 during more than 1 ns, thus indicating that the $AlCl_4^-$ polyanion tetrahedron should be very stable at 375 K.

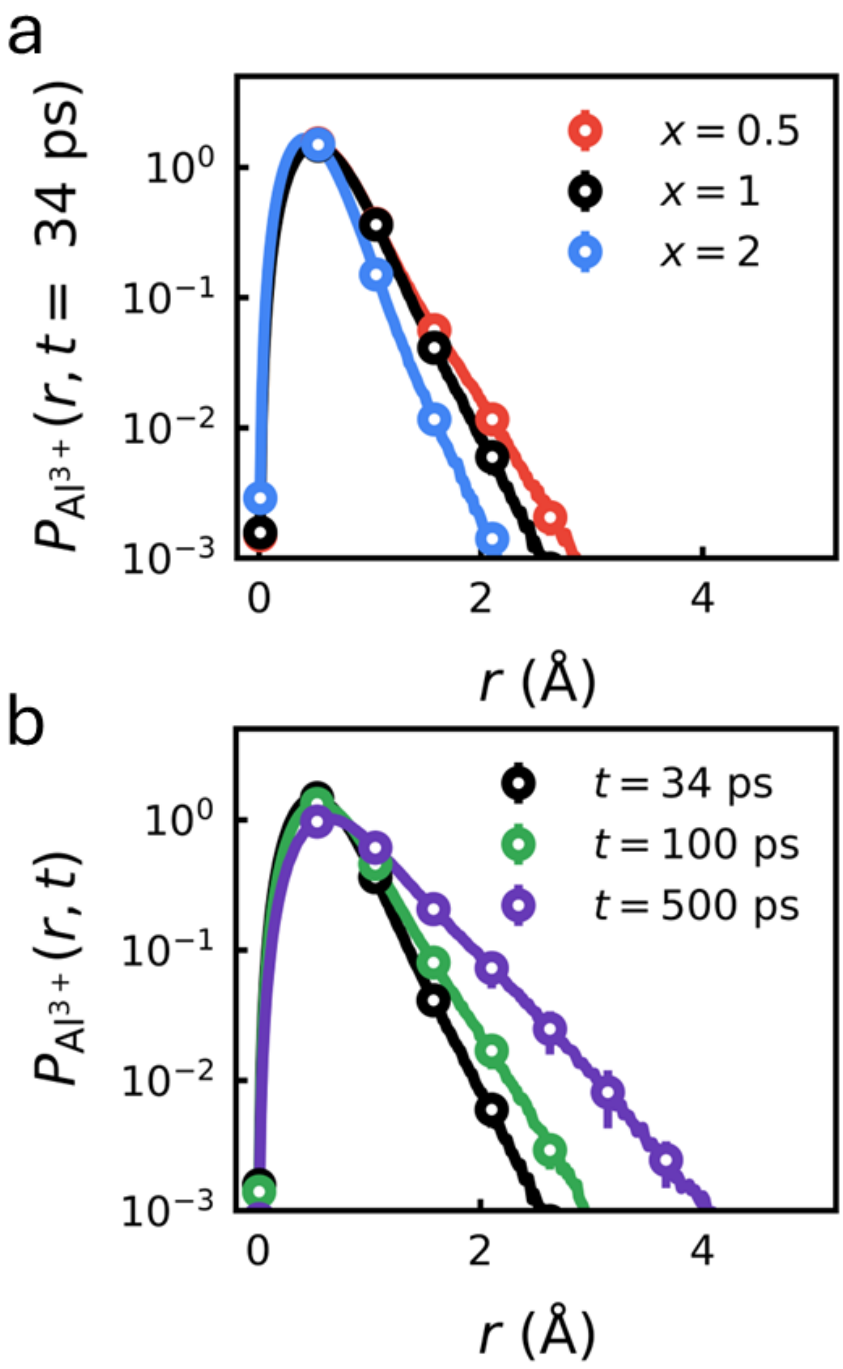

Supplementary Figure S2. The mobility of aluminum ion and polyanions. a. The self-part of van Hove correlation functions $P_{Al^{3+}}(r, t = 34\ \text{ps}) = 4\pi r^2 G_s(r, t = 34\ \text{ps})$ of aluminum ions in Li_xAlCl_{3+x} melts for different values of *x*. Unlike lithium ions, aluminum ions exhibit only rattling motions, as expected for solids. **b.** The self-part of van Hove correlation functions, $P_{Al^{3+}}(r, t) = 4\pi r^2 G_s(r, t)$ of aluminum ions in $LiAlCl_4$ for various timescales. Aluminum ions show only rattling behaviors even at longer times.

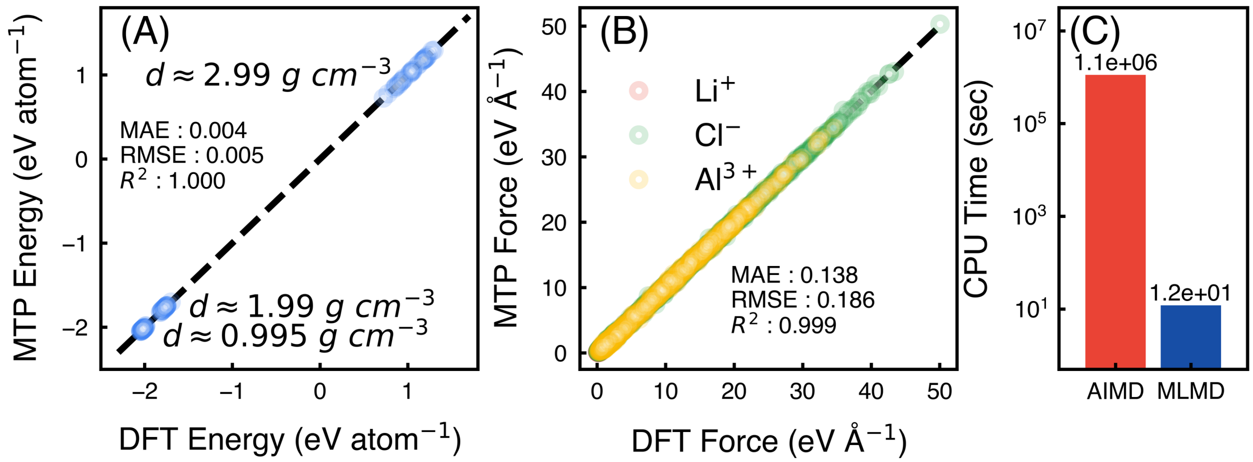

Supplementary Figure S3. Benchmark result and performance of trained MTP: a. The parity plot of energy calculated by MTP and DFT. Each point in the figure represents a potential energy value calculated from a configuration. Note that AIMD simulations are carried out three different densities (*d*'s). **b.** The parity plot of forces calculated by MTP and DFT. Each point in the figure represents a force acting on an ion within the configuration, distinguished by ion types. **c.** CPU Time required for AIMD and MLMD simulations for simulating 96 atom $LiAlCl_4$ at 1000 K at isothermal-isochoric condition.

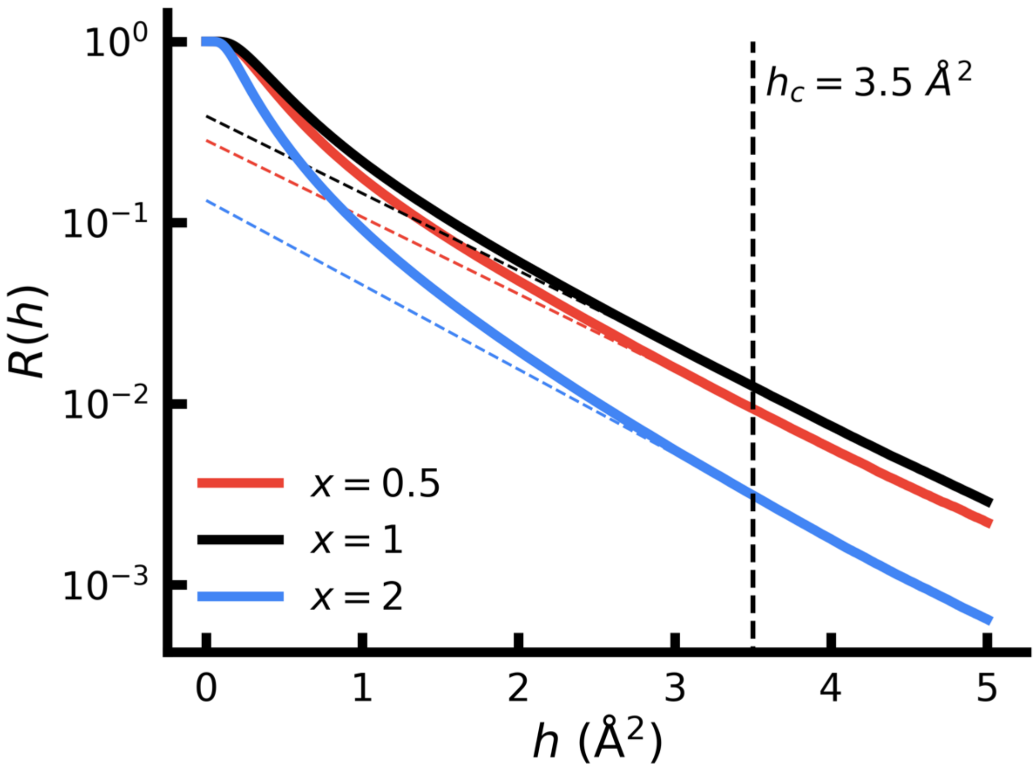

Supplementary Figure S4. Cumulative probability $R(h)$ of the hop function. The cumulative probability $R(h)$ is calculated to distinguish between the localized rattling motion (a caged state) and hopping motions (a hopping state) of lithium ions. $R(h)$ decreases rapidly up to $h = h^*$ and shows an exponential decay after $h > h^*$. Therefore, h^* is then a threshold between the independent hopping motion and a correlated rattling motion. We find that h^* depends on the composition (x) of Li_xAlCl_{3+x} melts. Therefore, we choose a sufficiently large value of $h = h^* = 3.5$ Å^2 as a criterion. Only when a lithium ion undergoes a hopping motion with $h > h^*$, we consider the motion as a hopping motion in this study.

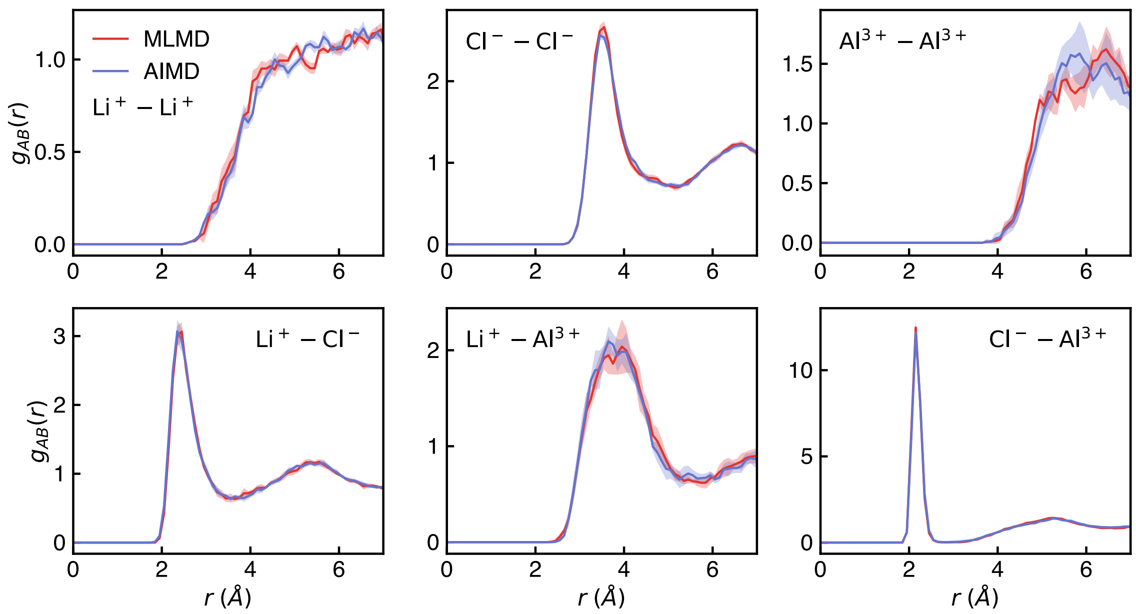

Supplementary Figure S5. Comparison of the radial distribution functions obtained from ab initio molecular dynamics simulations (AIMD) and machine learning molecular dynamics simulations (MLMD). The radial distribution functions ($g_{AB}(r)$'s) between A and B atomic species of $LiAlCl_4$. Red and blue lines correspond to $g_{AB}(r)$'s obtained from MLMD and AIMD simulations. Note that there is no significant difference in $g_{AB}(r)$'s between MLMD and AIMD simulations. To perform a systematic comparison, we employed the same system size for both MLMD and AIMD simulations. $g_{AB(r)}$'s obtained from MLMD simulations are almost identical to those from AIMD simulations.

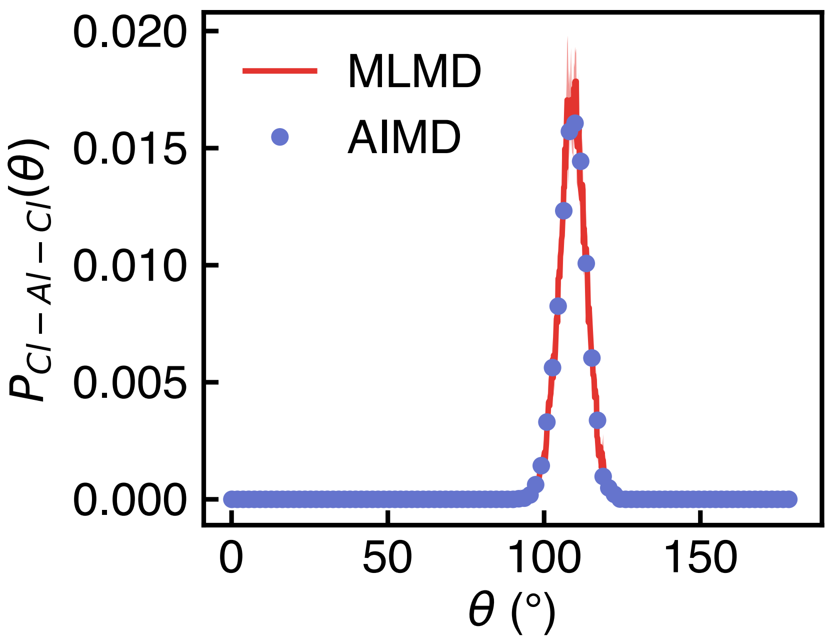

Supplementary Figure S6. Comparison of the angle probability distribution obtained from ab initio molecular dynamics simulations (AIMD) and machine learning molecular dynamics simulations (MLMD). The distribution ($P_{Cl-Al-Cl}(\theta)$) of the angle (θ) between two vectors that connect an aluminum ion and two chloride ions of tetrahedral polyanions by using both MLMD and AIMD simulations. $P_{Cl-Al-Cl}(\theta)$'s from MLMD and AIMD simulations are almost identical to each other with a prominent peak at θ = 109.5°.

Supplementary Equations

We calculate the radial distribution function ($g_{AB}(r)$) between the ion A and the ion B, defined as

$$g_{AB}(r) = \frac{V}{4\pi r^2 N_A N_B} \sum_i^{N_A} \sum_j^{N_B} \langle \delta(r - |\vec{r_i} - \vec{r_j}|) \rangle \quad \text{(S1)}$$

where V, N_A, N_B are the system volumes, the number of A, and the number of B, respectively. $\vec{r_i}$ denotes the position vectors of the ith atom. The result of $g_{AB}(r)$ for different kinds of ions is shown in Supplementary Fig. S2c.

We also define the cage correlation function, $C_{cage}(t)$, to quantify how stable a polyanion tetrahedron of $AlCl_4^-$ would be as follows,

$$C_{cage}(t) = \frac{\langle \vec{L_i}(t) \cdot \vec{L_i}(0) \rangle}{\langle \vec{L_i}(0) \cdot \vec{L_i}(0) \rangle}. \quad \text{(S2)}$$

Here, $\vec{L_i}(t) = [f(r_{ij})]$ is the neighbor vector of the ith Al^{3+} ion at time t and r_{ij} is the distance between the ith Al^{3+} ion and the jth Cl^- ion. $f(r_{ij})$ = 1 if $r_{ij} < r_c$ and 0 otherwise, where r_c of 3Å is the cutoff distance. If the ith Al^{3+} ion were to sustain its tetrahedron with an identical set of Cl^- ions during time t, $C_{cage}(t)$ would be 1. If all the Cl^- ions of the tetrahedron of the ith Al^{3+} ion were to be replaced by a new set of Cl^- ions, $C_{cage}(t) = 0$. The result of $C_{cage}(t)$ in $LiAlCl_4$ at 375 K is illustrated in Supplementary Fig. S2d.

Supplementary Notes

1. Parameters used for density functional theory (DFT) calculations for energy, force and stress

For the potential readers' convenience, all parameters are explained based on those provided by the VASP package, and parameters not mentioned were kept at their default values. For the equilibration and ab-initio molecular dynamics (AIMD) steps, the parameters listed below were used. The 'PREC' parameter, which indicates the precision related to energy cutoff, FFT grids, and the accuracy of the projectors in real space, was set to 'low'. Projection operators were evaluated in real space (LREAL = Auto). The plane wave energy cutoff (ENCUT) was set to 520 eV, and the global break condition for the electronic SC-loop (EDIFF) was set to 0.0012 eV. Gaussian smearing was applied with a width of 0.5 eV (ISMEAR = 0, SIGMA = 0.5), and symmetry was disabled (ISYM = -1). The 'ISIF' parameter was set to '2' to calculate forces and the stress tensor. In addition, a minimal Γ-centered 1x1x1 k-point mesh was used. Equilibration was performed for 5000 steps followed by 1000 steps of AIMD under isothermal-isochoric conditions at 1000 K, with a time step of 2 fs.

For the high-level DFT calculations, the optB88-vdW exchange-correlation functional with PBE correlation was employed, using the recommended parameters from VASP (PARAM1 = 0.1833333333, PARAM2 = 0.22) were employed. In this case, the 'PREC' parameter was set to 'Accurate'.

Note that we employ parameters of (EDIFF, SIGMA) = (0.0012 eV, 0.5) to maximize the efficiency of sampling phase space and obtaining training data via AIMD simulations. In order to check the accuracy of the AIMD simulations, we also perform AIMD simulations with (EDIFF, SIGMA) = (1.2×10^{-5} eV, 0.05). We find that all the total energy, temperature, and the radial distribution functions obtained with (EDIFF, SIGMA) = (0.0012 eV, 0.5) are almost identical within statistical errors to those with (EDIFF, SIGMA) = (1.2×10^{-5} eV, 0.05) (Figures S7 and S8).

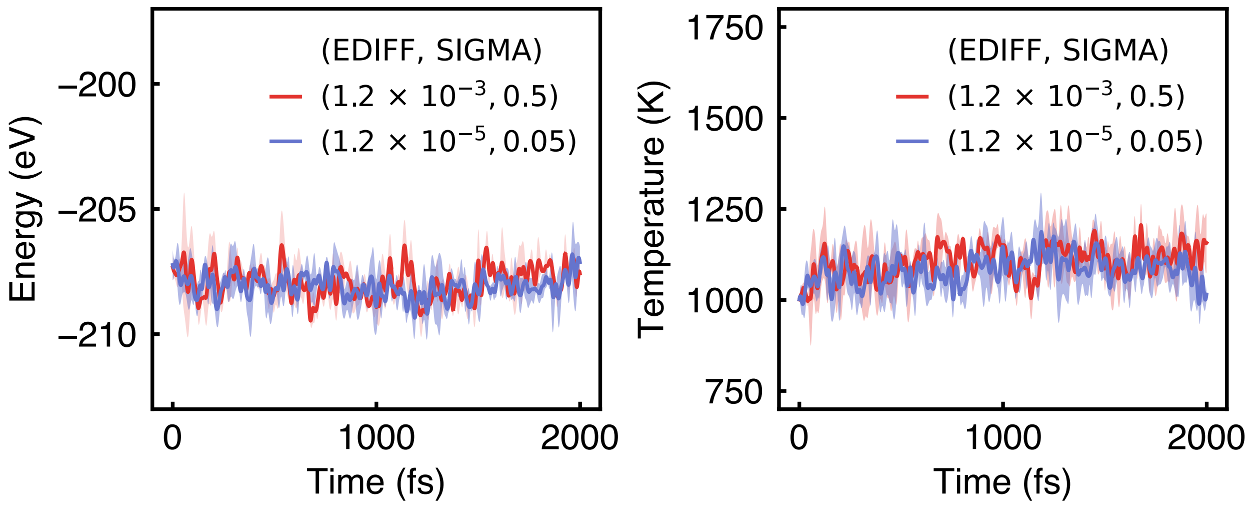

Supplementary Figure S7. Comparisons of AIMD simulation results with two different parameter sets. (a) The total energy of $LiAlCl_4$ as a function of time during 2 ps. (b) The system temperature of $LiAlCl_4$ as a function of time during 2 ps. Red and blue lines correspond to DFT calculations with (EDIFF, SIGMA) = (0.0012 eV, 0.5) and (1.2 × 10^{-5} eV, 0.05), respectively.

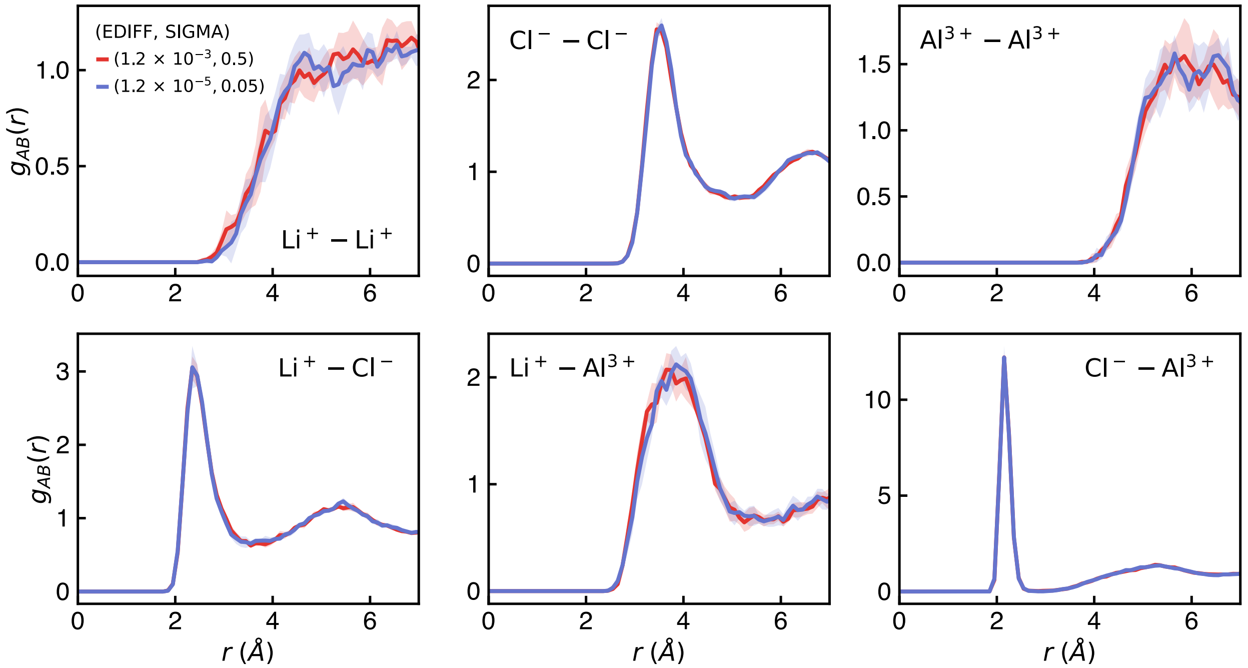

Supplementary Figure S8. Comparisons of AIMD simulations results with two different parameter sets. Radial distribution functions ($g_{AB}(r)$) between ions obtained from AIMD simulations. Red and blue lines correspond to DFT calculations with (EDIFF, SIGMA) = (0.0012 eV, 0.5) and (1.2 × 10^{-5} eV, 0.05), respectively.

2. Parameters used to construct MTP potential.

The parameters such as the maximum level of the basis function of MTP, the cutoff distance, and the weights on errors in energy, force, and stress were set according to the paper by Podryabinkin et al. (*J. Chem. Phys.* 159, 084112 (2023)) that introduced the development and improvement of the MLIP software package. The authors provided an example of a Cu crystalline system with 111 surface. They systematically trained MTPs with different maximum levels of basis functions− 8th, 12th, and 16th. The cutoff distance was set to 5.0 Å, and the extrapolation boundaries were defined as $\gamma_{save} = 2$, and $\gamma_{break} = 10$. As expected, the MTP with the 16th level showed the highest accuracy, with an energy root mean square error (RMSE) of 5 ± 2 meV/atom and a force RMSE of 68 ±1 meV/ Å. From the perspective of evaluating the MTP training performance based on these error values, the parameters (the 16th level) we used also yielded reasonable errors for solid electrolytes, with an energy RMSE of 5 meV/atom, and a force RMSE of 186 meV/Å.

3. MLMD simulation results for amorphous LPS ($75Li_2S•25P_2S_5$)

We construct the machine learning potential (MLP) for amorphous LPS ($75Li_2S•25P_2S_5$) systems by following the same procedure employed for Li_xAlCl_{3+x}. Then, we carry out MLMD simulations and hop function analysis at T = 300 K and density of 1.882 ± 0.002 g/cm^3.

Figure S9 depicts the structural properties of amorphous LPS system. As shown in a representative snapshot (Figure S9(a)), Li ions are dispersed well while P and S ions construct tetrahedral structures well. Radial distribution functions, especially between Li ions, are consistent with previous simulation studies (*Nat. Commun.* 11, 1483 (2020), *ACS Appl. Mater. Interfaces*, 16, 18874 (2024), *Chem. Mater*. 35, 891 (2023)) in the sense that $g_{Li^+-Li^+}(r)$'s have two split peaks in the range of r from 3 to 5 Å. The probability distribution function ($P_{S-P-S}(\theta)$) of the angle (θ) between two neighbor P–S bonds (that share a P atom of a tetrahedron) has a high peak at $\theta = 109.5°$, thus indicating that P and S ions form stable tetrahedrons in our simulations.

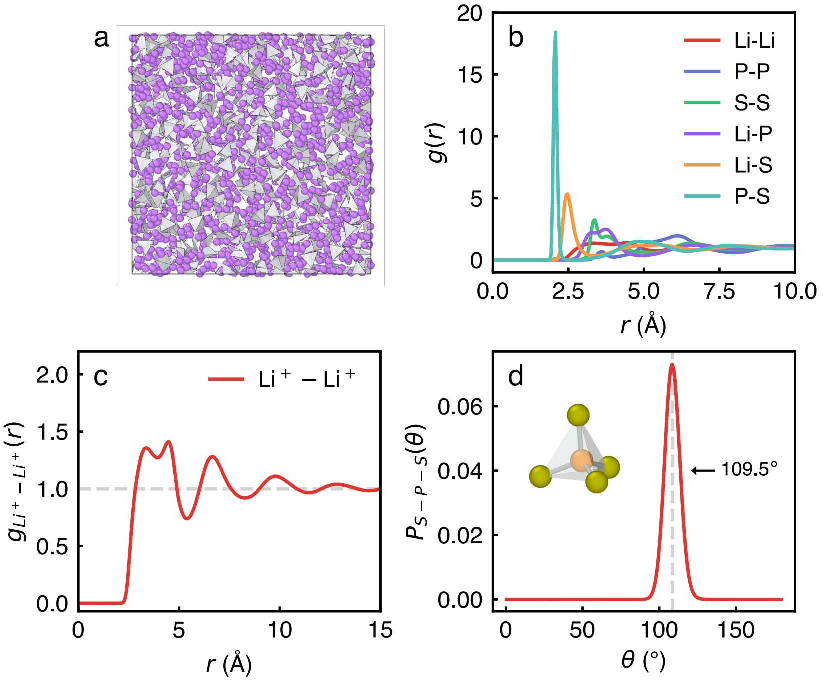

Supplementary Figure S9. Structural properties of amorphous LPS obtained from MLMD simulations. (a) A representative MLMD simulation snapshot of amorphous LPS system. (b) The radial distribution functions ($g_{AB}(r)$'s) between A and B atomic species of amorphous LPS systems. (c) The radial distribution functions ($g_{Li^+-Li^+}(r)$) between Li ions. (d) the probability

distribution function ($P_{S-P-S}(\theta)$) of the angle (θ) between two neighbor P–S bonds (that share a P atom) of tetrahedrons.
